# Biological and quantitative-SAR evaluations, and docking studies of (E)-N -benzylidenebenzohydrazide analogues as potential antibacterial agents

**DOI:** 10.17179/excli2016-388

**Published:** 2016-06-17

**Authors:** Mohammad Sayed Alam, Sefat Jebin, M. Mostafizur Rahman, Md. Latiful Bari, Dong-Ung Lee

**Affiliations:** 1 Division of Bioscience, Dongguk University, Gyeongju 780-714, Republic of Korea; 2 Department of Chemistry, Jagannath University, Dhaka 1100, Bangladesh; 3 Food analysis and Research laboratory, Centre for Advance Research in Sciences, University of Dhaka, Dhaka-1000, Bangladesh

**Keywords:** hydrazone Schiff base, antimicrobial activity, physicochemical properties, docking study

## Abstract

A series of 15 (*E*)-*N'*-benzylidenebenzohydrazide analogues were evaluated for their antimicrobial activities against eleven pathogenic and food-borne microbes, namely, *S. aureus *(G^+^),* L.* *monocytogenes *(G^+^), *B. subtilis *(G^+^), *K.* *pneumonia *(G¯), *C.* *sakazakii *(G¯), *C.* *freundii *(G¯), *S.* *enterica *(G¯), *S.* *enteritidis *(G¯), *E*. *coli *(G¯), *Y.* *pestis *(G¯), and *P. aeruginosa *(G¯). Most of the compounds exhibited selective activity against some Gram-negative bacterial strains*. *Of the compounds tested (**3a-o**), **3b** and **3g** were most active against *C.* *freundii* (MIC = ~19 µg mL^-1^). Whereas, compounds **3d, 3i, 3k **and** 3n** exhibited MIC values ranging from 37.5 to 75 μg mL^-1^ against *C.* *freundii*, and compounds **3e**, **3l** and **3n** had MIC values of ~75 μg mL^-1^ against* K. pneumonia*. Quantitative structure-antibacterial activity relationships were studied using physicochemical parameters and a good correlation was found between calculated octanol-water partition coefficients (clogP; a lipophilic parameter) and antibacterial activities. *In silico* screening was also performed by docking high (**3b** and **3g**) and low (**3n**) activity compounds on the active site of *E. coli* FabH receptor, which is an important therapeutic target. The findings of these *in silico* screening studies provide a theoretical basis for the design and synthesis of novel benzylidenebenzohydrazide analogues that inhibit bacterial FabH.

## Introduction

**Figure 1:**
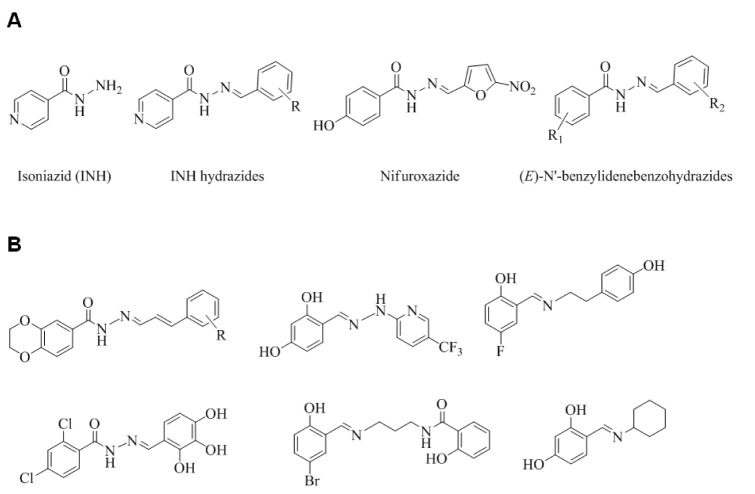
Structures of (A) key molecules and (B) some reported potential FabH inhibitors possessing a hydrazone Schiff base or Schiff base motif

Isoniazid (isonicotinylhydrazide; INH) (Figure 1A(Fig. 1)) is a front-line antimycobacterial that is usually used to treat tuberculosis. INH exerts its activity *via* a bacterial catalase-peroxidase (KatG) (Suarez et al., 2009[36]) by forming isonicotinic acyl-NADH complex, which interacts firmly to enoyl-acyl carrier protein reductase (InhA), and thus, inhibits the synthesis of mycolic acid, and mycobacterial cell growth. A hydrazone Schiff base analogue of INH (Figure 1A(Fig. 1)) has been reported to have antimycobacterial activity against INH resistant *M. tuberculosis *strains and to exhibit lower toxicity than INH (Sah and Peoples, 1954[31]; Bavin et al., 1954[7]) and nifuroxazide - a commercial hydrazide-hydrazone Schiff base antibiotic used to treat colitis and diarrhea. Another analogue of nifuroxazide was found to show antimicrobial activity comparable to that of ceftriaxone (Rollas et al., 2002[30]). Currently, bacterial resistance to existing drugs, such as, β-lactam antibiotics, macrolides, quinolones, and vancomycin, is considered one of the most important health concerns (Cassell and Mekalanos, 2001[9]), and thus, sustained research efforts are being made to identify new drugs with wide therapeutic windows, broad spectrum activities, and novel modes of action. A literature survey revealed that synthetics and natural products containing the hydrazone Schiff base or Schiff base motif exert their antibiotic activities by inhibiting fatty acid biosynthesis (FAB) (Cheng et al., 2009[10]; Shi et al., 2010[32]; Song et al., 2014[35]). In prokaryotic organisms, FAB is crucially required for cell viability and growth (Song et al., 2014[34]). β-Ketoacyl-acyl carrier protein (ACP) synthase III (FabH or KAS III) plays an important role in bacterial FAB (Khandekar et al., 2003[17]) and is considered an essential functional enzyme in the bacterial FAB system. Bacterial FabH has no homolog in man, and thus, is an attractive target for the design of new antibiotics (Lee et al., 2009[21] and 2012[22]). The structures of several reported antibacterial FabH inhibitors that possess a Schiff base or hydrazone Schiff base motif are shown in Figure 1B(Fig. 1), and interestingly, these structural motifs are also found in many bioactive compounds. 

Hydrazone Schiff bases possess -CONHN=CH- moiety and they are widely exploited to syntheses of various biologically active molecules. In addition, they have shown wide-ranging bioactivities. For example, antimicrobial (Vijesh et al., 2013[38]), anticancer (Dandawate et al., 2012[11]), anti-mycobacterial (Mahajan et al., 2011[23]), anti-convulsant (Kulandasamy et al., 2009[18]), anti-inflammatory (Rajitha et al., 2011[29]), analgesic (Kümmerle et al., 2009[19]), anti-hypertensive (Leal et al., 2012[20]), anti-platelet (Mashayekhi et al., 2013[24]), and anti-protozoal activities (Carvalho et al., 2012[8]). Hydrazones are also used as herbicides, insecticides, nematocides, rodenticides, plant growth regulators, and housefly sterilants (Akelah et al., 1993[1]; Mohan et al., 1988[25]). Hydrazone Schiff bases are versatile polydentate chelating agents and form a variety of complexes with various transition and inner transition metals with different bioactivities and practical applications (Gad et al., 2000[15]; Zhong et al., 2007[40]; Fan et al., 2010[14]; Juliano et al., 2010[16]). These wide-ranging biochemical activities and uses of hydrazone Schiff bases and their complexes have attracted considerable research attention. 

During our continued studies (Alam et al., 2013[6], 2014[4], 2015[5]) on novel bioactive compounds, we evaluated the antimicrobial activities of a series of (*E*)-*N*'-benzylidenebenzohydrazide analogues (**3a**-**o**) exhibiting structural similarities with INH and nifuroxazide. In a previous study, we described the synthesis and cytotoxic and antioxidant activities of a series of fifteen (*E*)-*N*'-benzylidenebenzohydrazide analogues (Alam and Lee, 2016[3]). Here, we report the *in vitro* antibacterial activities of these fifteen (*E*)-*N*'-benzylidenebenzohydrazide analogues (**3a**-**o**) against 11 pathogenic and food-borne bacterial strains, that is, three Gram-positive (*Staphylococcus aureus*,* Listeria* *monocytogenes*, and *Bacillus subtilis*), and eight Gram-negative bacterial strains (*Klebsiella* *pneumoniae*, *Cronobacter* *sakazakii*, *Citrobacter* *freundii*, *Salmonella* *enterica*, *Salmonella* *enteritidis*, *E*. *coli*, *Yersinia* *pestis*, and *Pseudomonas aeruginosa*). In addition, physicochemical characteristics of the synthesized compounds were used to access quantitative structure-antibacterial activity relationships (Q-SAR). Finally, *in silico* screening was performed using docking simulations using the X-ray crystallography determined structure of *E*. *coli* FabH to investigate the binding affinities and interaction modes of benzylidene-hydrazone analogues at its active site. Computer-assisted drug design (CADD) was used is a useful tool for conducting docking simulations, and provides important information regarding the natures of interactions, favorable bioactive conformations, and binding affinities of ligands at active sites of target receptors (Alam and Lee, 2016[3]). For these reasons, this technique is helpful for identifying therapeutic lead compounds (Shoichet et al., 2002[33]).

## Material and Methods

### Chemistry

**Figure 2:**
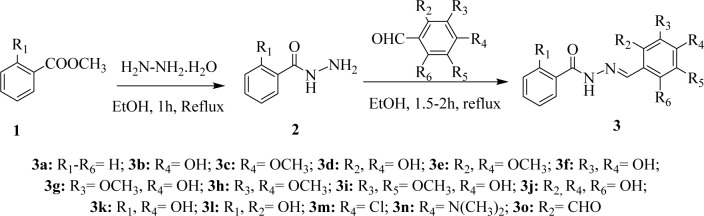
The synthesis of novel (*E*)-N'-benzylidenebenzohydrazide analogues 3a-o

The **(***E*)-*N*'-Benzylidenebenzohydrazide analogues (**3a**-**o**) examined in the present study were prepared as we previously described (Alam and Lee, 2016[3]) from their corresponding benzohydrazides (**2**) as presented in Figure 2(Fig. 2). The mixture of methyl esters of benzoic acid (13.6 g, 0.1 M) or salicylic acid (15.2 g, 0.1 M) and hydrazine hydrate (12.5 g, 0.25 M) in 100 mL ethanol (roughly) were refluxed for 3 h. TLC method was used to observe the reaction progress. The obtained solid products were separated and purified by recrystallization using aqueous ethanol. The structures of the synthesized compounds were characterized by comparing the previously reported physical and spectral data. The benzohydrazides (**2**, 1 mM) so obtained were then refluxed with suitably substituted benzaldehydes (1 mM) in ethanol for 1.5-2.5 h. The reaction progress was monitored by TLC. At room temperature, the reaction mixtures give solid crude products, and they were filtered and crystallized using ethanol to afford the pure compounds (**3a**-**o**) (81-96 % yield).

### Antibacterial screening 

A previously reported filter paper disc diffusion method (Alam and Lee, 2010[2]) was used to determine the *in vitro* antibacterial effects of **3a**-**o** against eleven bacterial strains. Nutrient agar (NA) media (Difco Laboratories, Lawrence, KS), a bacterial growing medium was inoculated with liquid cultures (0.2 mL) of the microorganisms. Discs socked with test samples (**3a**-**o**) were kept on pre-treated agar Petri dishes and incubated aerobically at 37^ o^C (24 h). DMSO and nalidixic acid were used as negative and positive controls, respectively. Bactericidal activity was defined as inhibitory zones diameters in mm. Evaluations were performed in triplicate. 

Minimal inhibitory concentrations (MIC, in μg/mL) against *Klebsiella* *pneumonia *(JCM 1662) and *Citrobacter* *freundii* (JCM 1657) were determined using the serial dilution technique (Alam and Lee, 2010[2]) using nutrient broth medium (DIFCO). MIC was defined as the lowest concentration of the tested compound (in DMSO) that inhibited bacterial growth. 

### Computational analysis

The molecular geometries of the (*E*)-*N*'-benzylidenebenzohydrazide analogues (**3a**-**o**) were examined using standard bond length and angles using the ChemBio3D ultra Ver. 14 molecular modeling program (CambridgeSoft Corporation, Cambridge, MA 02140, USA). Physicochemical properties were calculated using Molinspiration Cheminformatics Software (Molinspiration Cheminformatics, SK 90026 Slovensky Grob, SR). The method used for calculating clogP values was developed by Molinspiration (miLogP2.2-2005) derived from the group contributions of more than twelve thousand drug-like compounds, and the theoretical logP and experimental logP values were used as a training set for fitting correction factors. The molecular polar surface are as (PSAs) were obtained as sums of fragment contributions (Ertl et al. 2000[13]). Molecular lipophilicity potentials (MLPs) and PSAs maps were analyzed using Molinspiration Galaxy 3D Structure Generator (ver. 2013.02 beta).

### Docking studies

The molecular geometries of compounds **3c**, **3i** and **3j** were constructed using standard bond lengths and angles as mentioned above, and energy minimized using the Hartree-Fock method at 6-31G basis set with R-Closed-Shell wave function in the ChemBio3D Ultra Ver. 14.0 software (GAMESS Interface). The docking studies were performed using crystal structure of *E. coli* FabH-CoA complex retrieved from the Protein Data Bank (PDB code: 1HNJ). To prepare the receptor for docking studies, co-crystallized ligand and water molecules were removed, while polar hydrogen atoms and Kollman-united charges were included to the FabH receptor molecule. The necessary pdb and pdbqt files of ligands and *E. coli* FabH receptor were prepared using AutoDock 4.2 software (Morris et al., 2009[26]). The study was carried out using the usual docking protocol applied for AutoDock Vina in PyRx 0.8 software (Trott and Olson, 2010[37]) where free rotation was allowed through single bonds of ligands. The docking results were analyzed using Discovery Studio 4.0 (Accelrys, Inc. San Diego, CA 92121, USA) and binding scores were calculated using iGEMDOCK software (Yang and Chen, 2004[39]).

## Results and Discussion

### Antibacterial activities

Compounds **3a-o** were evaluated for their *in vitro *antibacterial activities against three Gram-positive bacteria, *S. aureus*,* L.* *monocytogenes*, and *B. subtilis*, and eight Gram-negative bacteria *K.* *pneumonia*, *C.* *sakazakii*, *C.* *freundii*, *S.* *enterica*, *S.* *enteritidis*, *E*. *coli*, *Y.* *pestis*, and *P.* ***aeruginosa*** by disc diffusion. As presented in Table 1(Tab. 1), compound **3e** inhibited the growths of all bacterial strains except *S.* *enterica*, but its activity was relatively weak. A half of the tested compounds inhibited G(-)-bacteria *K.* *pneumonia*, *C.* *freundii*, and *E*. *coli,* but only **3e** inhibited G(+)-bacteria. Of the compounds tested, **3b** and **3g** were most effective against *C.* *freundii*, and had activities similar to nalidixic acid (the positive standard). Compounds bearing an OH group at the R_4_-position (*para*-position) in the benzylidene phenyl ring (e.g., **3b**, **3g** > **3d** > **3i** > **3k**) better inhibited *C.* *freundii*. Whereas, compounds with additional OH substitution at the *meta*-position (e.g. **3f**) or *ortho*-positions (e.g. **3j**) were devoid of activity.

The minimal inhibitory concentrations (MICs) of selected compounds were determined against *K. pneumonia* and *C. freundii*; results are summarized in Table 2(Tab. 2). Compounds **3b** and **3g** had the lowest MIC values (18.75 μg/mL), followed by **3d**, **3i,** and **3k** (37.5 μg/mL) against *C. freundii*, whereas compounds **3e, 3l, **and** 3n** had similar MIC values (75 μg/mL) against *K. pneumonia*. Above results indicate the presence of a polar hydroxyl group at the R_4_-position (*para*-position) favors activity, but that *meta* positioned OH groups reduce activity.

### Quantitative-SAR study

To explain quantitative structure-antibacterial activity relationships (Q-SAR) of the fifteen (*E*)-*N*'-benzylidenebenzohydrazide analogues (**3a-o)**, physicochemical calculations were conducted using ChemBio3D Pro 12 molecular modeling (CambridgeSoft Corporation, Cambridge, MA 02140, USA) and Molinspiration Cheminformatics software (Molinspiration Cheminformatics, SK 90026 Slovensky Grob, SR). The physicochemical properties of molecules, such as, their lipophilicities and polar surface areas (PSAs) play important roles in determining biological responses, and are commonly used to study the structure-activity relationships of bioactive molecules in medicinal chemistry (Alam et al., 2013[6]; Desai et al., 2014[12]). These parameters are now well-accepted major experimental and theoretical tools for drug design and discovery. The physiochemical parameters of all synthesized compounds are listed in Table 3(Tab. 3). 

**Figure 3:**
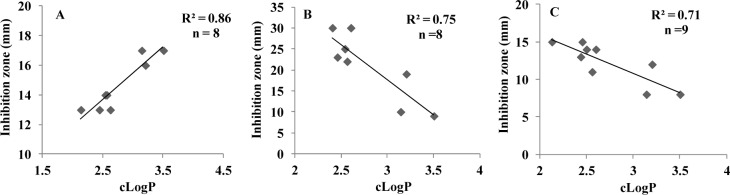
Correlations between inhibition zones (mm) and calculated octanol-water partition coefficients (clogP) of active (*E*)-N'-benzylidenebenzohydrazide analogues against (A) *K.* *pneumonia *(3b, 3d-g, 3k, 3l and 3n), (B) *C.* *freundii *(3b, 3d, 3e, 3g, 3i, 3k, 3l and 3n), and (C) *E*. *coli *(3b, 3d-g, 3i, 3k, 3l and 3n).

**Figure 4:**
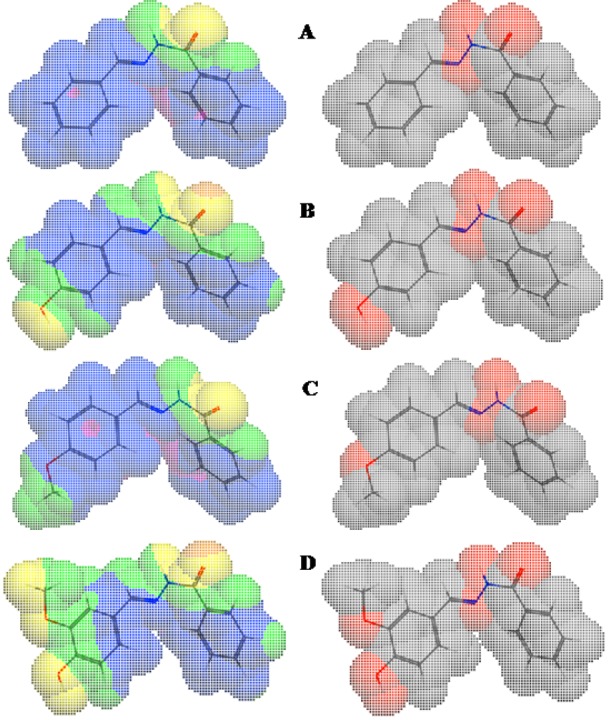
Molecular lipophilicity potentials (left) and polar surface areas (right) of 3a (A), 3b (B), 3c (C), and 3g (D) showing areas of lipophilicity (blue), intermediate lipophilicity (pink), hydrophilicity (yellow), intermediate hydrophilicity (green), nonpolar (gray/white), and polar (red) areas.

In the present study, a considerable number of compounds were found to be active against *K.* *pneumonia*, *C.* *freundii*, and *E*. *coli*. It is well-known that the octanol-water partition coefficient (logP) of a molecule depends on its hydrophobicity and polarity, which facilitate transit across cellular membranes. Therefore, we examined the correlation between the inhibitory effects of **3a-o** against *K.* *pneumonia*, *C.* *freundii*, and *E*. *coli* and their clogP values. The correlation coefficients (*r*^2^) between clogP values and the inhibitory potencies of active molecules against *K.* *pneumonia*, *C.* *freundii*, and *E*. *coli* were; 0.86 (n = 8; **3b**, **3d**-**3g**, **3k**, **3l** and **3n**), 0.75 (n = 8; **3b**, **3d**,**3e**, **3g**, 3i, **3k**, **3l**, and **3n**), and 0.71 (n = 9; **3b**, **3d**-**3g**, **3i**, **3k**, **3l**, and **3n**), respectively. Although only a relatively small number of compounds were examined, significant correlations were observed, whereby antibacterial activity increased with clogP against *K.* *pneumonia* but decreased with clogP against *C.* *freundii* and *E*. *coli* (Figure 3(Fig. 3)). However, the above correlations should be treated with caution because the clogP values of **3a, 3c, 3h, 3m, **and** 3o **fell within the medium range for active compounds, but they were in fact inactive. Therefore, we compared maps of lipophilicity potential (MLP) and PSA of two selected active and inactive compounds (**3b **and** 3g **vs. **3a** and **3c**). It was found that lipophilicity potentials and the polar surface areas of these two pairs of compounds differed (Figure 4(Fig. 4)). These results suggest that the distributions of more hydrophilic and polar areas at the *para*-position of benzylidene phenyl ring importantly determine activity against specific bacterial strains.

### Molecular docking studies

β-Ketoacyl-(acyl-carrier-protein) synthase III (FabH), a key bacterial enzyme plays a crucial regulatory role in bacterial fatty acid synthesis (FAS) by initiating fatty acid elongation cycles and providing feedback regarding the regulation of FAS, which is essentially required for prokaryotic cell metabolism, viability, and growth. However, the bacterial FAS pathway differs considerably from in man, and bacterial FabH proteins are highly conserved at the sequence and structural level and show no significant homology with any human protein. In addition, the active site residues of FabH receptor are common for Gram-positive and -negative bacterial strains (Nie et al., 2005[27]). These features make FabH protein a potential therapeutic target for the design of novel and broad-spectrum antimicrobial agents as selective and nontoxic FabH inhibitors. To predict the binding affinities of hydrazone Schiff base analogues to FabH, molecular docking of (*E*)-*N'*-benzylidenebenzohydrazide analogues having high (**3b** and **3g**)and low (**3n**) activity with the active site of *E. coli *FabH receptor was performed. The crystal structure of *E. coli* FabH-CoA complex (Qiu et al., 2001[28]) was retrieved from the Protein Data Bank (PDB ID: 1HNJ) and compounds **3b**, **3g**, and **3n** were docked at its active site using a standard docking protocol. *In silico* docking results are presented in Table 4(Tab. 4).

**Figure 5:**
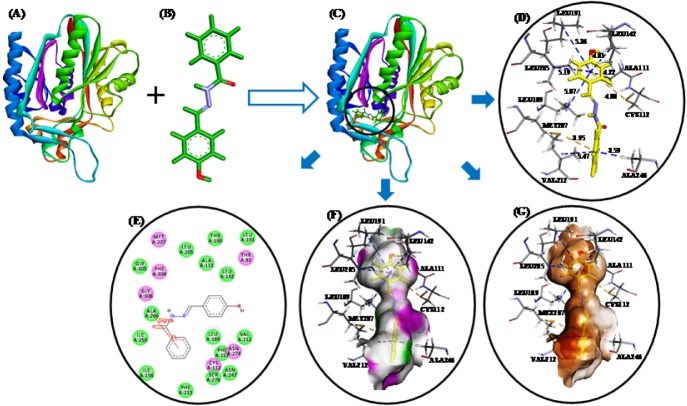
(A)* E*. *coli* FabH receptor (PDB ID: 1HNJ). (B) HF/6-31G optimized geometry of ligand 3b. (C) Docked ligand-receptor complex (the circle shows the ligand binding site) (D) Binding model of 3b with *E*. *coli* FabH; π-alkyl and π-sulfur interactions are shown by *blue* and *yellow broken lines*, respectively. (E) 2D view of interacting essential amino acid residues at the ligand binding site; The residues involved in electrostatic and covalent interactions are shown by *purple circles* and amino acids involved in van der Waals interactions are shown by *green circles*. (F) Showing hydrogen bond donor (pink) and acceptor (green) surfaces. (G) Showing hydrophobic interactions (brown) surfaces.

**Figure 6:**
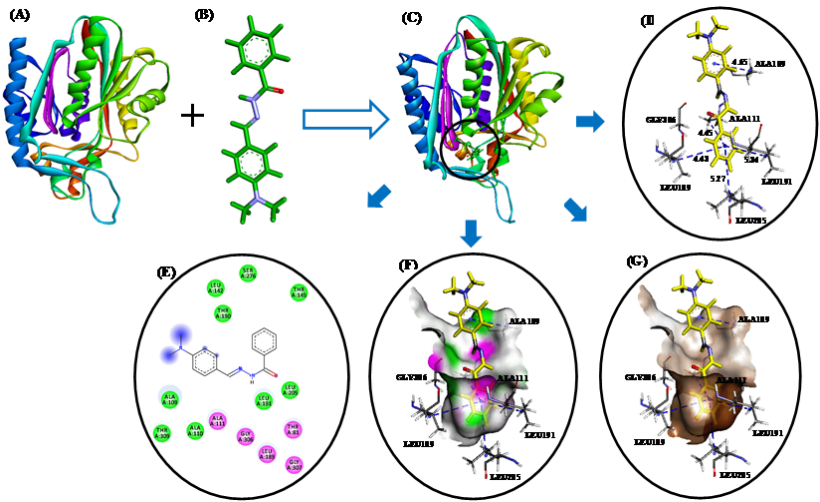
(A)* E*. *coli* FabH receptor (PDB ID: 1HNJ). (B) HF/6-31G optimized geometry of ligand 3n. (C) Docked ligand-receptor complex (the circle indicates the ligand binding site) (D) Binding model of compound 3n with *E*. *coli* FabH; H-bond and π-alkyl interactions are shown by *red *and* blue broken lines*, respectively. (E) 2D view of interacting essential amino acid residues at the ligand binding site; The residues involved in electrostatic and covalent interactions are shown by *purple circles* and amino acids involved in van der Waals interactions are shown by *green circles*. (F) Showing hydrogen bond donor (pink) and acceptor (green) surfaces. (G) Showing hydrophobic interactions (brown) surfaces.

These studies revealed that all three compounds bind to the same active site of FabH receptor as endogenous malonyl-CoA ligand and other reported hydrazone and Schiff base FabH inhibitors (Song et al., 2014[35]; Zhou et al., 2013[41]; Shi et al., 2010[32]). The binding modes and the different types of interactions found for compound **3b** (most active) and **3n** (least active) were compared and are shown in Figures 5(Fig. 5) and 6(Fig. 6), respectively. To measure the affinity of ligands for the receptor, we analyzed binding scores for compounds **3b**, **3g**, and **3n** by iGEMDOCK software (Yang and Chen, 2004[39]); results are shown in Table 4(Tab. 4). According to docking scores, the high activity compounds **3b** and 3g had higher binding affinities with FabH receptor than **3n** (the binding energies of **3b**, **3g** and **3n** were -80.36, -84.33, and -72.22 kcal/mol, respectively). Regarding total binding energies, van der Waal contributions for 3b and 3g were -71.29 and 71.02 kcal/mol, respectively, and H-bond contributions were -9.07 and 12.41 kcal/mol, respectively. While for 3b only van der Waal interactions (-72.22 kcal/mol) contributed to total binding energy. In the binding model of **3b** and *E*. *coli* FabH receptor, two π-sulfur interactions were observed between the benzoyl-phenyl ring and Met207 (3.95 Å) and between the benzylidene-phenyl ring and Cys112 (4.88 Å), together with π-alkyl interactions with Ala111(4.22 Å), Leu142 (4.81 Å), Leu189 (5.07 Å), Leu191 (5.36 Å), Leu205 (5.19 Å), Val212(5.47 Å), and Ala246 (3.59 Å). However, in the binding model of **3g** with *E*. *coli* FabH receptor, a H-bond between the carbonyl group of Leu189 (C...HO: 2.96 Å) and methoxy proton, and π-sulfur and π-sigma interactions of Met207 (3.83 Å) and Ala246 (2.59 Å), respectively were observed including π-alky interactions with Ala111 (3.84 Å), Cys112 (5.32 Å), Leu142 (5.07 Å), Leu189 (5.08 Å), and Leu191 (5.48 Å). Whereas in the binding model of **3n** with *E*. *coli* FabH receptor, no H-bond, π-sulfur, or π-sigma interactions were observed, although π-alkyl interactions with Ala109 (4.65 Å), Ala111 (4.45 Å), Leu189 (4.43 Å), Leu191 (5.34 Å), Leu205 (5.27 Å), and Gly306 (4.43) involving benzoyl- and benzylidene-phenyl rings were observed. In addition, the following van der Waal's interactions were also observed: between **3b** and Cys112, Ile156, Phe157, Thr190, Met207, Phe213, Ile250, and Gly305; between **3g** and Ile156, Phe157, Leu205, Val212, Phe213, Asn247, Ala246, Ile250, Ser276, Gly305, and Gly307; and between **3n** and Ala110, Leu142, Thr145, Thr190, Ser276, and Thr309. These results indicate compounds **3b**, **3g**, and **3n** bind to the same active site of FabH receptor. However, the most active compounds **3b** (Figure 5(Fig. 5)) and **3g** (figure not shown) attached more tightly in the active site of FabH receptor due to additional H-bond, π-sulfur, π-sigma, and hydrophobic interactions, which resulted in higher binding affinities than compound **3n** (Figure 6(Fig. 6)).

## Conclusion

The present study reports the antibacterial activities of a series of 15 (*E*)-*N*'-benzylidenebenzohydrazide analogues against eleven pathogenic and food-borne bacterial strains, of these eleven, three were Gram-positive, i.e., *S. aureus*,* L.* *monocytogenes*, and *B. subtilis*, and eight were Gram-negative, i.e., *K.* *pneumoniae*, *C.* *sakazakii*, *C.* *freundii*, *S.* *enterica*, *S.* *enteritidis*, *E*. *coli*, *Y.* *pestis*, and *P. aeruginosa*. The compounds subjected to antibacterial assay, **3b **and** 3g**, which are containing one OH group at the *para*-position of the benzylidene phenyl ring, showed lowest MIC values against *C.* *freundii*. Physicochemical calculations indicated that the antibacterial activities of (*E*)-N'-benzylidenebenzohydrazide analogues (**3b**, **3d**-**3g**, **3i**, **3k**, **3l**, and **3n**) against *K.* *pneumonia*, *C.* *freundii*, and *E*. *coli* correlated well with calculated logP values. *In silico* screening using molecular docking studies were performed to predict the binding affinities and modes of interaction of the most active (**3b** and **3g)** and least active (**3n)** compounds to the active site of *E*. *coli* FabH receptor. Compounds **3b **and** 3g** were found to bind effectively to the active site of *E*. *coli* FabH receptor with high affinity, suggesting their potentials as FabH inhibitors. We believe the above findings can be used to facilitate the design and synthesis of novel benzylidenehydrazone analogues as potential antibacterial as FabH inhibitors.

## Figures and Tables

**Table 1:**
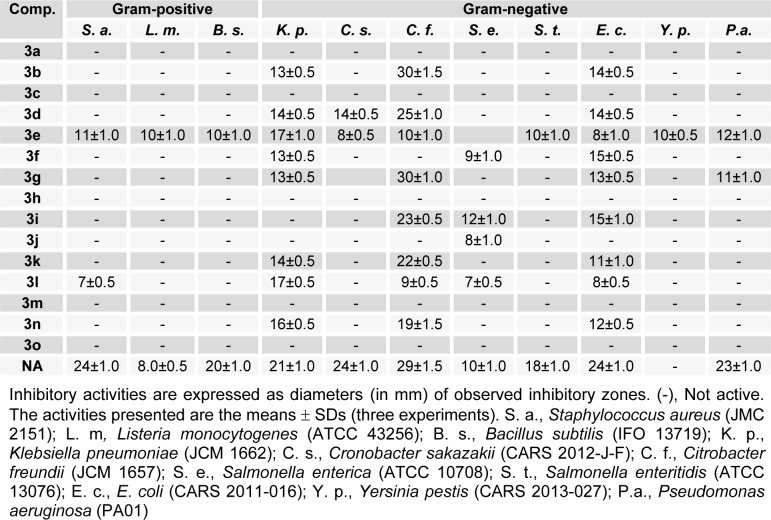
*In* *vitro* antimicrobial profiles of (*E*)-N'-benzylidenebenzohydrazide analogues (3a-o) as determined by growth inhibition zones

**Table 2:**
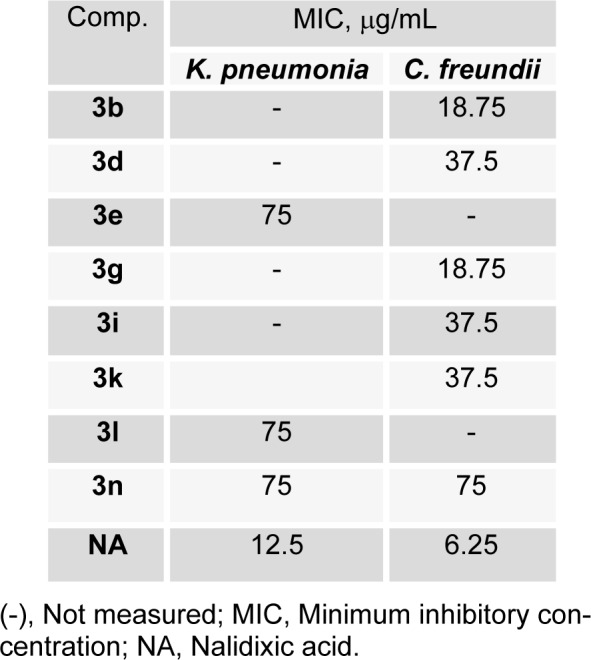
MIC of some (E)-N'-benzylidenebenzohydrazide analogues derivatives against selected bacterial strains

**Table 3:**
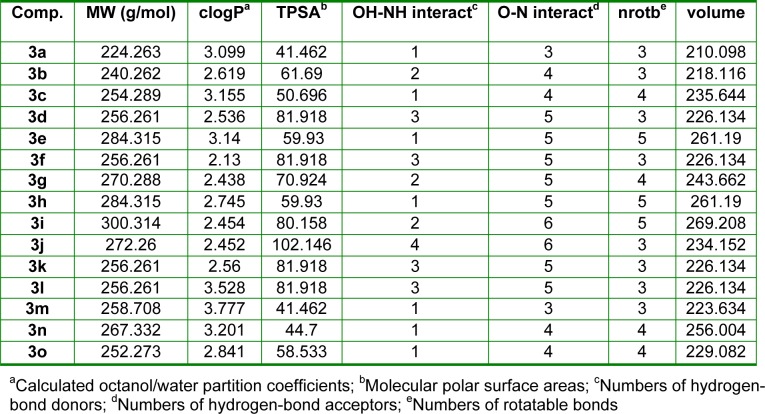
Molinspiration calculations of the molecular properties of (E)-N'-benzylidenebenzohydrazide analogues (3a-o)

**Table 4:**
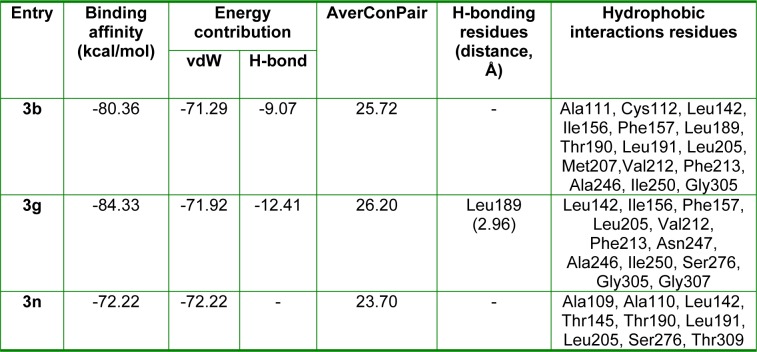
Docking energies and molecular interactions of the ligand molecules (3b, 3g, and 3n) with *E. coli *FabH receptor
